# Employing Cationic Kraft Lignin as Additive to Enhance Enzymatic Hydrolysis of Corn Stalk

**DOI:** 10.3390/polym15091991

**Published:** 2023-04-23

**Authors:** Jingliang Xu, Huihua Li, Md. Asraful Alam, Gul Muhammad, Yongkun Lv, Anqi Zhao, Shen Zhang, Wenlong Xiong

**Affiliations:** 1 School of Chemical Engineering, Zhengzhou University, Zhengzhou 450001, China; 2 Henan Center for Outstanding Overseas Scientists, Zhengzhou 450001, China; 3 School of Life Sciences, Zhengzhou University, Zhengzhou 450001, China

**Keywords:** kraft lignin, cationic modification, crosslinking modification, enzymatic hydrolysis, additives

## Abstract

A water-soluble cationic kraft lignin (named JLQKL_50_), synthesized by combining quaternization and crosslinking reactions, was used as an additive to enhance the enzymatic hydrolysis of dilute-alkali-pretreated corn stalk. The chemical constitution of JLQKL_50_ was investigated by Fourier transform infrared spectroscopy, ^1^H nuclear magnetic resonance (NMR) and ^13^C NMR spectroscopy, and elemental analysis. The enzymatic hydrolysis efficiency of corn stalk at solid content of 10% (*w*/*v*) was significantly improved from 70.67% to 78.88% after 24 h when JLQKL_50_ was added at a concentration of 2 g/L. Meanwhile, the enzymatic hydrolysis efficiency after 72 h reached 91.11% with 10 FPU/g of cellulase and 97.92% with 15 FPU/g of cellulase. In addition, JLQKL_50_ was found capable of extending the pH and temperature ranges of enzymatic hydrolysis to maintain high efficiency (higher than 70%). The decrease in cellulase activity under vigorous stirring with the addition of JLQKL_50_ was 17.4%, which was much lower than that (29.7%) without JLQKL_50_. The addition of JLQKL_50_ reduced the nonproductive adsorption of cellulase on the lignin substrate and improved the longevity, dispersity, and stability of the cellulase by enabling electrostatic repulsion. Therefore, the enzymatic hydrolysis of the corn stalk was enhanced. This study paves the way for the design of sustainable lignin-based additives to boost the enzymatic hydrolysis of lignocellulosic biomass.

## 1. Introduction

Over the past few decades, significant economic growth around the world has increased the demand for energy and chemicals derived from renewable resources because of the limited sources of fossil fuels and their serious environmental problems [1,2]. In this context, lignocellulosic biomass (LCB) has been regarded as the most promising source of renewable fuel and chemicals and a potential material to reduce the global reliance on fossil fuels [3,4]. LCB is the most abundant renewable non-grain feedstock for sugar production and contains essential platform molecules to produce a wide variety of fuels and chemicals by fermentation or chemical processing [5,6,7]. One of the mandatory steps in the biorefinery of LCB is the enzymatic hydrolysis of pretreated LCB to produce sugar syrups. The economic feasibility of the biorefinery of LCB is highly restricted by the efficiency of enzymatic hydrolysis [8].

Considerable effort has been devoted to improving the efficiency of enzymatic hydrolysis [9,10]. The following strategies are often used to enhance the efficiency of the enzymatic hydrolysis of lignocellulose: (1) using pretreatment methods to overcome the obstinacy of lignocellulose biomass [11]; (2) adding beneficial additives during enzymatic hydrolysis; (3) screening microorganisms with high cellulase production rates; (4) using enzymes prepared with activities complementary to cellulase; (5) regulating the composition of lignocellulose through genetic engineering; (6) conducting batch feeding to offset the limited mass transfer at high solid content [12]. The application of additives to enhance enzymatic hydrolysis has become of great interest owing to its high operational feasibility and ability to reduce the non-productive binding of cellulase to lignin in substrates [13]. Additives, including metal ions (e.g., Fe^3+^ and Co^2+^) [14], proteins (e.g., bovine serum albumin, soybean protein, casein, etc.) [15], and various types of surfactants or polymers (e.g., polyethylene glycol, tween, saponin, glycolipid, sophora resin, cetyltriethylammnonium bromide, sodium dodecyl sulfate, lignin derivatives, etc.) [16,17,18], have been used to boost enzymatic hydrolysis. Lignin in lignocellulose cannot be completely removed, although residues in the pretreated raw materials negatively impact enzymatic hydrolysis through steric hindrance and unproductive cellulase adsorption. Water-soluble lignin used as an additive can effectively promote the enzymatic hydrolysis of lignocellulose [19]. By modifying lignin with appropriate functional groups, it has the ability to reduce the original negative effects of lignin on enzymatic hydrolysis [20]. Meanwhile, this is conducive to the value-added utilization of abundant and renewable lignin. Thus, lignin-based additives for enzymatic hydrolysis have received considerable attention [19,20,21,22,23].

Generally, lignin with poor water solubility (e.g., alkali lignin from the paper and pulp industry and enzymatic hydrolysis lignin from biorefinery) inhibits or only slightly promotes enzymatic hydrolysis [20]. Water-soluble sodium lignosulfonate (LS) obtained from sulfite pulping can effectively increase the sugar content in the enzymatic hydrolysis system of lignocellulose. When the ratio of LS with a suitable degree of sulfonation and molecular weight to substrate lignin was 1:1, it had the best effect on the efficiency of enzymatic hydrolysis [21]. This is because LS can increase the negative charge of cellulase to reduce the ineffective adsorption of cellulase on substrate lignin with a negative charge. In addition, other types of water-soluble lignin used as additives for enzyme hydrolysis have been reported. Lin et al. synthesized water-soluble lignin polyoxyethylene ether (KL-PEG) and found that when adding 2 g/L KL-PEG, the enzymatic hydrolysis efficiency of eucalyptus pretreated with dilute acid increased from 58.3% to 93.8%, and the cellulase activity remained at 44% after 72 h of enzymatic hydrolysis [22]. In another study, Lai et al. synthesized a lignin-based surfactant by grafting polyethylene glycol diglycidyl ether onto organic solvent lignin and found that the surfactant could adsorb on the substrate lignin faster than cellulase, thus promoting the enzymatic hydrolysis efficiency of lignocellulose by increasing the effective adsorption of cellulase on the substrate [23]. These studies have suggested that the strategy of synthesizing water-soluble lignin is of great significance to the enhancement of enzymatic hydrolysis.

In this study, a new water-soluble lignin-based polymer was synthesized using kraft lignin (KL) as a raw material. The KL was first modified by a cationization reaction using 3-chloro-2-hydroxypropyltrimethylammonium chloride and then crosslinked by using poly(ethylene glycol) diglycidyl ether. The aim of this modification method was to enable electrostatic repulsion by causing both the cellulase and substrate lignin to become electropositive after the modified lignin was adsorbed onto them. This strategy is different from those of previous studies, which aimed to increase the negative charge or the hydrophilicity of both cellulase and substrate lignin [19,20,21,22,23]. This modified lignin (named JLQKL_50_) was investigated by Fourier transform infrared spectroscopy (FT-IR), elemental analysis, nuclear magnetic resonance (NMR), and zeta potential analysis. JLQKL_50_ was added as an additive to the enzymatic hydrolysis system, and the effect of JLQKL_50_ on the enzymatic hydrolysis efficiency of dilute-alkali-pretreated corn stalk was studied. The mechanism of JLQKL_50_ affecting the enzymatic hydrolysis of lignocellulose was also investigated. This study enriches the strategies to design lignin-based additives to enhance the enzymatic hydrolysis of lignocellulosic biomass.

## 2. Materials and Methods

### 2.1. Materials and Chemicals

Corn stalk crushed and screened with a 40–60 mesh sieve was used as the substrate for enzymatic hydrolysis after intensive drying. Cellulase was obtained from Azure Biological Co., Ltd. (Qingdao, China). Cellulase filter paper activity was 55.65 FPU/g, and its protein content was 89.47 mg/mL, found according to the method reported elsewhere [24]. KL was separated from black liquor via an acid precipitation method. The black liquor produced from the kraft pulping process of softwood was supplied by Tiger Forest & Paper Group Co., Ltd. (Yueyang, China). The acid precipitation method included the following processes: firstly, the black liquor was heated to 50 °C under stirring and the pH value of the black liquor was adjusted to 2 using aqueous sulfuric acid (20 wt%); secondly, the produced suspension was continuously stirred for 2 h at 50 °C; finally, the KL powder was obtained by suction filtration, washing with deionized water, and intensive drying in an oven at 50 °C.

In addition, 3-chloro-2-hydroxypropyltrimethylammonium chloride (CHPTMAC) solution (60 wt%) and poly(ethylene glycol) diglycidyl ether (PEGDGE) were purchased from Sigma-Aldrich Trading Co., Ltd. (Shanghai, China). Sulfuric acid (H_2_SO_4_, 95–98 wt%) and sodium hydroxide (NaOH, 96 wt%) were supplied by Luoyang Chemical Reagent Factory (Luoyang, China). Acetic acid (CH_3_COOH) and sodium acetate (CH_3_COONa) were purchased from Shanghai Macklin Biochemical Technology Co., Ltd. (Shanghai, China).

### 2.2. Synthesis of JLQKL_50_

JLQKL_50_ was synthesized by a two-step modification reaction of KL, in which the quaternization method as reported in the literature was used [25,26,27]. First, a quantitative 25% solution of KL was weighed in a four-necked flask and heated up to 85 °C in a water bath (HH-W0-5L, China), and CHPTMAC was added dropwise using a peristaltic pump, where the mass ratio of KL to CHPTMAC was 2:1. A quantitative 50% sodium hydroxide solution was added to maintain the pH of the reaction above 11 for 5 h. The obtained and cooled QKL_50_ solution was weighed in a four-necked flask and heated up to 45 °C with a water bath. PEGDGE was added to the flask dropwise by using a peristaltic pump, where the mass ratio of QKL_50_ to PEGDGE was 10:1 and the reaction was conducted for 4 h. The obtained JLQKL_50_ solution was purified by dialysis using a dialysis bag with a cut-off molecular weight of 1000 Da, concentrated under reduced pressure and then freeze-dried. The reaction equation for the synthesis of JLQKL_50_ is shown in Figure S1 (Supporting Information).

### 2.3. Enzymatic Hydrolysis of Corn Stalk

The pretreatment of corn stalk was performed according to the literature [28]. The corn stalk was accurately weighed into a four-necked flask, and 2% (*w*/*v*) NaOH solution was added to make the solid–liquid ratio 1:20, and it was placed in a water bath at 80 °C for 2 h. After pretreatment, the solid was separated from the liquid using a filtration method, which was performed with a ceramic Büchner funnel (200 mm), suction flask (Sichuan Shubo (Group) Co., Ltd., Chongzhou, China), and recirculating water vacuum pump (Zhengzhou Great Wall Technology Industry and Trade Co., Ltd., Zhengzhou, China). The residue was washed with deionized water to a neutral pH and dried in an oven at 60 °C. Component analysis of the pretreated corn stalk was performed according to the method of the National Renewable Energy Laboratory [29], and the cellulose content of the pretreated corn stalk was found to be 63.19%, hemicellulose content 11.17%, and acid insoluble lignin 6.15%.

Pretreated corn stalk (2 g) was placed in a 100 mL blue-capped bottle, and 20 mL of acetic acid–sodium acetate buffer at pH 4.8, cellulase with 10 FPU/g of substrate, and 2 g/L additive were added successively. No additive was added to the control group. The blue-capped bottles were placed in a shaker (IS-RDS6T, Suzhou Jiemei Electronic Co., Ltd., Suzhou, China) at 50 °C and 200 rpm for enzymatic hydrolysis. At different intervals, such as 6, 12, 24, 48, and 72 h, 200 µL of each sample was collected during the reaction, centrifuged at 10,000 r/min for 10 min, and diluted 10,000 times, and then the glucose content was detected by ion chromatography. Enzymatic hydrolysis efficiency data were obtained from triplicate readings.

### 2.4. Analysis and Characterizations

The glucose concentrations in the enzymatic hydrolysis products were analyzed by ion chromatography on a Dionex™ CarboPac™ PA20 column with an injection volume of 25 µL and an eluent of 200 mmol/L sodium hydroxide solution and ultrapure water at a flow rate of 0.5 mL/min. The temperatures of the column and detector were maintained at 30 °C.

The enzymatic hydrolysis efficiency was calculated by the following equation:

Y_C_ = (C_g_ × V × 0.9)/m_c_ × 100%

where Y_C_ is the enzymatic hydrolysis efficiency, C_g_ is the glucose concentration, V is the buffer volume, m_c_ is the mass of cellulose in the pretreated corn stalk, and 0.9 is the conversion factor between cellulose and glucose.

An organic elemental analyzer (Vario Micro, Elementar Analysensysteme GmbH, Frankfurt, Germany) was used in quantifying the percentages of carbon, hydrogen, nitrogen, and sulfur elements in lignin samples.

An FT-IR system (Tensor II, Bruker Optics, Ettlingen, Germany) was used to analyze the functional groups of lignin samples.

The lignin samples were analyzed by liquid NMR (AVANCE NEO 400 MHZ, Bruker, Karlsruhe, Germany) and superconducting solid NMR (AVANCE (3) 400 WB, Bruker, Zurich, Switzerland).

The zeta potential of lignin samples at different pH values was measured with a nanoparticle size and zeta potential analyzer (DLS) (Zetasizer Nano ZS90, Malvern Panalytical, Spectris, Shanghai, China). The measurement of the average size of cellulase in water with or without additive was also conducted on this instrument.

## 3. Results and Discussion

### 3.1. Structural Characterizations of KL and JLQKL_50_

#### 3.1.1. FT-IR Spectra and Elemental Analyses of KL and JLQKL_50_

**Figure 1:**
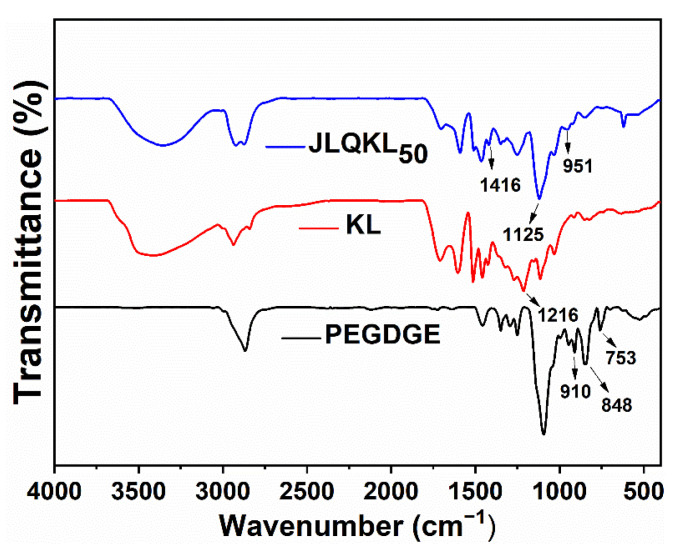
FT-IR spectra of KL, PEGDGE, and JLQKL_50_.

The FT-IR analysis results of KL, PEGDGE, and JLQKL_50_ are presented in Figure 1. Compared with the spectrum of KL, the spectrum of JLQKL_50_ showed the characteristic peak of a C–N bond at 1416 cm^−1^, and the stretching vibration peak of the alcoholic hydroxyl group at 1125 cm^−1^ was significantly enhanced. By contrast, the stretching vibration peak of the phenolic hydroxyl group at 1216 cm^−1^ was weakened [30]. These results suggested that the phenolic hydroxyl group in the lignin molecule was the reaction site for graft quaternization. The characteristic peaks at 753, 848, and 910 cm^−1^ corresponded to the epoxy group in PEGDGE and disappeared in JLQKL_50_ [31]. In addition, a new C–O–C stretching vibration peak at 951 cm^−1^ appeared in JLQKL_50_ [32], indicating the presence of the PEGDGE fragment in JLQKL_50_.

Table S1 (Supporting Information) shows the elemental compositions of KL and JLQKL_50_. The nitrogen content increased from 0.28% for KL to 2.165% for JLQKL_50_. The results indicated that quaternary ammonium groups were introduced to the lignin molecule, increasing the nitrogen content.

#### 3.1.2. ^1^H NMR and ^13^C NMR Analyses of KL and JLQKL_50_

**Figure 2:**
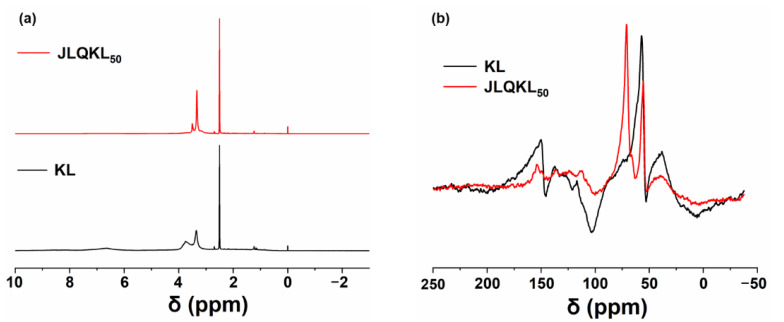
(**a**) ^1^H NMR and (**b**) ^13^C NMR of KL and JLQKL_50_.

The ^1^H-NMR spectra obtained for KL and JLQKL_50_ are illustrated in Figure 2a. In the spectrum of KL, the signal peaks in the 8.0–6.0 ppm range could be attributed to the phenolic hydroxyl group proton on lignin and almost disappeared in the spectrum of JLQKL_50_, indicating the involvement of the phenolic hydroxyl group of the lignin molecule in the reaction [33]. The strong peaks between 3.8 and 3.5 ppm are attributed to the methyl proton (–CH_3_) in lignin [33]. The chemical shifts of these two peaks were significantly enhanced in the spectrum of JLQKL_50_ after the introduction of quaternary ammonium groups on the lignin molecule. The sharp peak at 2.5 ppm was the solvent peak (DMSO-d6) [34,35].

Solid-state ^13^C NMR is a widely used method for the investigation of the lignin structure. In this study, it was used to analyze the chemical structures of KL and JLQKL_50_. As seen in Figure 2b, all the characteristic peaks in the spectrum of KL were retained in the spectrum of JLQKL_50_. For example, a peak at 55 ppm was observed in both the KL and JLQKL_50_ spectra, which was the characteristic methoxy group of lignin [36]. In contrast to KL, JLQKL_50_ exhibited a strong peak at 70 ppm, which was the O–C–C–O repeating unit contained in the PEGDGE crosslinker and indicated an effective crosslinking reaction between QKL_50_ and PEGDGE [37]. These results confirmed the successful synthesis of JLQKL_50_.

#### 3.1.3. Zeta Potential versus pH of KL and JLQKL_50_

The zeta potential of KL and JLQKL_50_ solutions under different pH conditions is presented in Figure S2 (Supporting Information). The zeta potential of KL was negative within the pH range of 3–12 because of the absence of positively charged functional groups in the molecule. In JLQKL_50_, an isoelectric point (pH = 7.5) was observed, which was attributed to the introduction of quaternary ammonium groups that could neutralize the original negatively charged groups in the KL molecule. When the pH increased from 3 to 7, the zeta potential of JLQKL_50_ gradually decreased because the level of ionization of the carboxyl group was increasing [38]. At a pH range of 7–9, the zeta potential slowly decreased because of the ionization of the unreacted phenolic hydroxyl groups. At the pH range of 9–12, the zeta potential stabilized when the phenolic hydroxyl groups were completely ionized [39].

### 3.2. Effects of Different Concentrations of Additive on Enzymatic Hydrolysis

**Figure 3:**
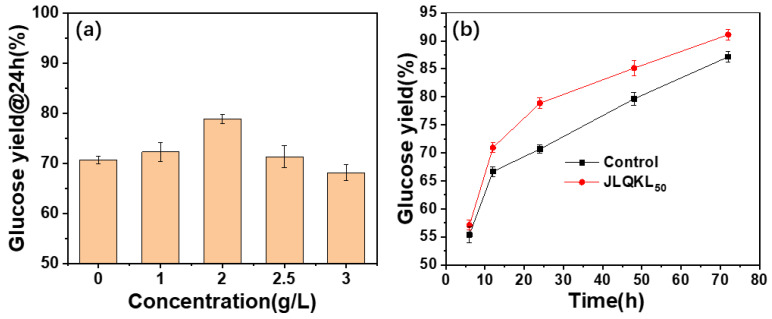
(**a**) Effect of different concentrations of additive on enzymatic hydrolysis efficiency. (**b**) Enzymatic hydrolysis efficiency at the optimum concentration 2 g/L.

Figure 3a presents the influence of the JLQKL_50_ concentration on the enzymatic hydrolysis efficiency of the corn stalk. The enzymatic hydrolysis efficiency increased with the additive concentration from 0 g/L to 2 g/L and then decreased. When the concentration of additive JLQKL_50_ was 2 g/L, the maximum enzymatic hydrolysis efficiency reached 78.88%, which was increased by 11.62% compared to that without the additive. When the concentration increased beyond 2 g/L, the enzyme activity was inhibited by excessive JLQKL_50_, resulting in a decrease in enzymatic hydrolysis efficiency [40]. Therefore, 2 g/L of additive was found to be the optimum.

Figure 3b depicts the enzymatic hydrolysis efficiency of the control and JLQKL_50_ from 6 to 72 h. The enzymatic hydrolysis efficiency of corn stalk at 6, 12, 24, 48, and 72 h increased to 57.10%, 70.94%, 78.88%, 85.13%, and 91.11% after the addition of 2 g/L of JLQKL_50_. The increase rate was 3.14%, 6.66%, 11.62%, 6.89%, and 4.56%, respectively. The enzymatic hydrolysis of corn stalk was obviously promoted at 24 h and the increase rate was the highest. Thus, we conducted subsequent experiments to check the environmental applicability of enzymatic hydrolysis with or without JLQKL_50_ based on the enzymatic hydrolysis efficiency at 24 h. This could help to improve the efficiency of experiments.

### 3.3. Environmental Applicability of JLQKL_50_-Enhanced Enzymatic Hydrolysis

**Figure 4:**
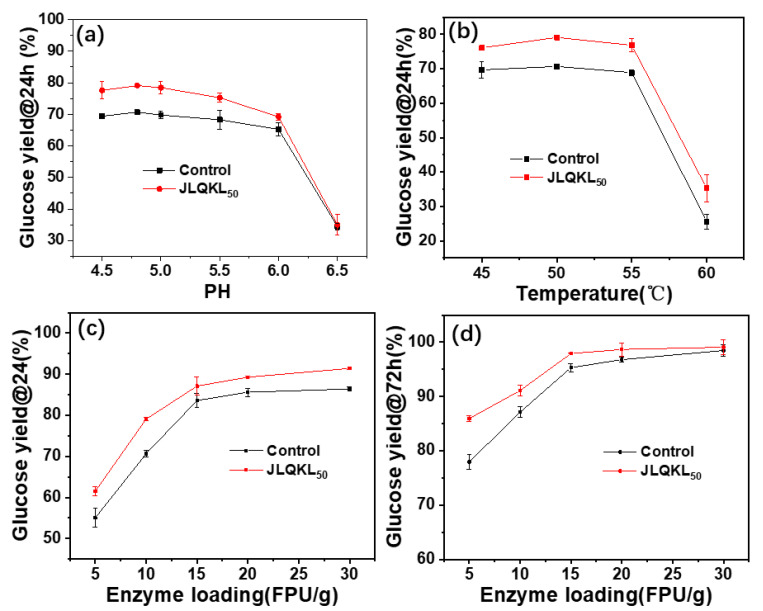
Enzymatic hydrolysis efficiency of corn stalk under different (**a**) buffer pH, (**b**) reaction temperatures, and (**c**,**d**) enzyme loading.

The effect of JLQKL_50_ on the enzymatic hydrolysis efficiency of corn stalk under the different buffer pH conditions is presented in Figure 4a. The enzymatic hydrolysis efficiency of the corn stalk tended to be stable when the buffer pH was 4.5–5.0 without additive. A decrease in the enzymatic hydrolysis efficiency of corn stalk was observed as the pH increased from 5.0 to 6.0. As the pH increased to values over 6.0–6.5, the enzymatic hydrolysis efficiency of corn stalk decreased rapidly to 35.04% because of the partial inactivation of cellulase at high buffer pH values. The addition of JLQKL_50_ did not change the trend of the enzymatic hydrolysis efficiency with the pH value. However, the enzymatic hydrolysis efficiency of corn stalk with the addition of JLQKL_50_ at pH 6.0 was even higher than that of the control at pH 4.8. This indicated that JLQKL_50_ contributed to widening the pH range for enzymatic hydrolysis to maintain high efficiency. This is possibly because the addition of JLQKL_50_ reduces the nonproductive adsorption of cellulase on substrate lignin by enabling electrostatic repulsion after JLQKL_50_ adsorbs on the cellulase and substrate lignin. As shown in Figure 4a, the decrease in the enzymatic hydrolysis efficiency of the control experiment was slightly slower than that of the experiment with the addition of JLQKL_50_ when the pH increased from 4.8 to 6.0. This demonstrated that the decrease in enzymatic hydrolysis efficiency with the addition of JLQKL_50_ was not only due to the decrease in cellulase activity. In fact, the positive electricity of JLQKL_50_ declined (Figure S2, Supporting Information) when the pH increased from 4.8 to 6.0, resulting in a reduction in the electrostatic repulsion between JLQKL_50_-adsorbed cellulase and substrate lignin. Hence, the ability of JLQKL_50_ in reducing the nonproductive adsorption of cellulase on substrate lignin dropped. These analyses indicated that the contribution of JLQKL_50_ in widening the pH range for enzymatic hydrolysis might be highly related to the ability of JLQKL_50_ in reducing the nonproductive adsorption.

The effect of JLQKL_50_ on the enzymatic hydrolysis efficiency of corn stalk at different temperatures is presented in Figure 4b. After the addition of JLQKL_50_ within a temperature range of 45–55 °C, the enzymatic hydrolysis efficiency significantly improved. The maximum enzymatic hydrolysis efficiency reached 78.88% at 50 °C with JLQKL_50_, which was much higher than that (70.67%) without the additive. The enzymatic hydrolysis efficiency was reduced significantly as the temperature increased above 55 °C, owing to the partial inactivation of cellulase under high temperatures. It could be seen that the enzymatic hydrolysis efficiency of corn stalk with the addition of JLQKL_50_ at 45 °C was even higher than that of the control at 50 °C. This implied that the addition of JLQKL_50_ could broaden the temperature range for enzymatic hydrolysis to maintain high efficiency.

The effect of enzyme loading on the enzymatic hydrolysis efficiency of corn stalk after 24 h was investigated, as shown in Figure 4c. In the absence of additive, the enzymatic hydrolysis efficiency increased rapidly and then slowly with increasing enzyme loading. The enzymatic hydrolysis efficiency of corn stalk improved at different loadings of the enzyme in the presence of the additive (JLQKL_50_). A 15 FPU/g of enzyme loading was required to increase the enzymatic hydrolysis efficiency of corn stalk without the additive to over 80%. Similar efficiency could be obtained by using 10 FPU/g enzyme loading and using JLQKL_50_ as the additive. The results showed that when the enzymatic hydrolysis efficiency of corn stalk reached ~80%, the addition of JLQKL_50_ reduced the enzyme loading by 33.33%. There are some studies reporting the ability of additives to effectively reduce the enzyme loading during the enzymatic hydrolysis process [41,42,43,44]. In addition, it could be found that the enzymatic hydrolysis time of 24 h was not sufficient to reach higher enzymatic hydrolysis efficiency than 95%, although the enzyme loading increased to 30 FPU/g. Thus, we further studied the effect of enzyme loading on the enzymatic hydrolysis efficiency of corn stalk after 72 h. The results are shown in Figure 4d. An enzyme loading of 15 FPU/g should be used to obtain higher enzymatic hydrolysis efficiency than 95% after 72 h. The enzymatic hydrolysis efficiency at 72 h with the addition of JLQKL_50_ reached 97.92%.

Here, we do not compare the results of the increase rate with those in previously published papers. This is because the enzymatic hydrolysis efficiency of the control experiments in our study was high, as shown in Figure 3b. In this study, the enzymatic hydrolysis efficiency of dilute-alkali-pretreated corn stalk after 72 h reached 87.14% under the following conditions: substrate solid content of 10% (*w*/*v*), 10 FPU/g of cellulase, pH 4.8, 50 °C, 200 rpm, and no additives. Therefore, the maximum improvement was only 12.86%, regardless of the used additive. The addition of JLQKL_50_ strongly improved the enzymatic hydrolysis efficiency of dilute-alkali-pretreated corn stalk according to our results. The increase rate was not very significant, only due to the good enzymatic hydrolysis efficiency of the control experiments. It is possible that the JLQKL_50_ additive also can present remarkable improvements in the enzymatic hydrolysis efficiency in a system in which the enzymatic hydrolysis efficiency of the control experiments is not very high.

### 3.4. Effect of Stirring on Cellulase Activity

**Figure 5:**
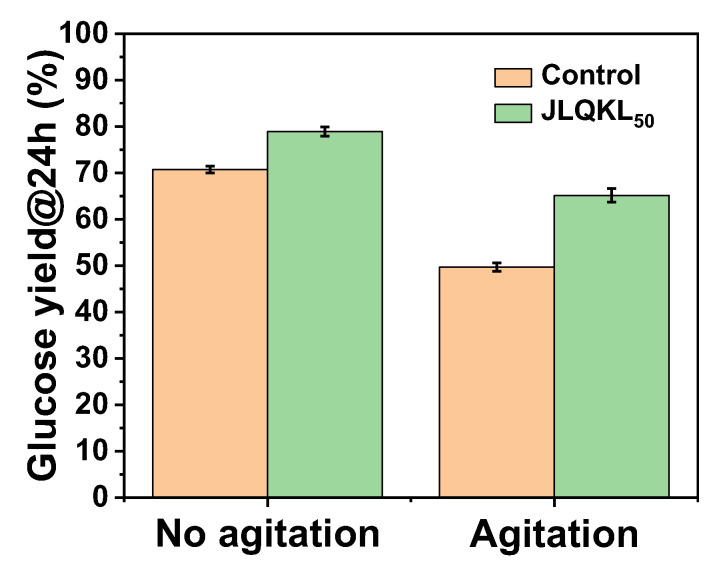
Effect of JLQKL_50_ on cellulase activity after intense agitation.

The cellulase and additive were added sequentially to the buffer and subjected to a temperature of 50 °C and agitation at 200 rpm for 72 h. Then, corn stalk was added for 24 h of enzymatic hydrolysis. The effect of JLQKL_50_ on cellulase activity after strong agitation was investigated. As shown in Figure 5, the enzymatic hydrolysis efficiency of cellulase without agitation and without an additive was 70.67%, whereas that of cellulase without an additive but with agitation was reduced to 49.70%. The decrease rate was 29.7%. Similarly, the efficiency of cellulase with the additive (JLQKL_50_) but without agitation was 78.88% and it was reduced to 65.14% after the addition of JLQKL_50_ and with agitation for 72 h. The decrease rate was 17.4%. The results showed that cellulase was easily deactivated after strong agitation when no additive was added and presented high activity when an additive (JLQKL_50_) was added, which was beneficial to the recycling of cellulase with sufficient activity.

### 3.5. Effect of JLQKL_50_ on the Aggregation and Dispersion of Cellulase

**Figure 6:**
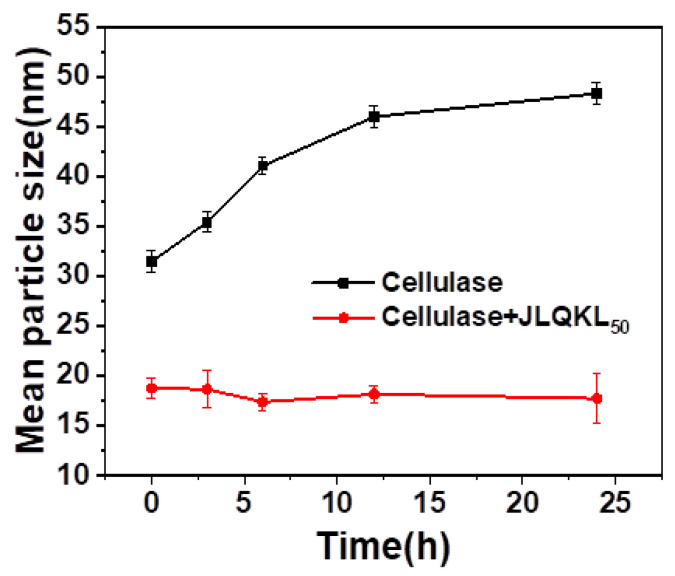
Effect of storage time on the mean diameter of cellulase in water.

Dynamic light scattering (DLS) analysis was used to determine the average size of cellulase in water. The results of the mean diameter versus storage time are shown in Figure 6. The initial mean diameter of cellulase was ~31 nm. With the increasing storage time, the mean diameter of cellulase increased gradually and approached ~48 nm after storage for 24 h. This indicated that the aggregation of cellulase increased with storage time. Meanwhile, in the presence of JLQKL_50_, the initial mean diameter of cellulase decreased to ~18 nm and remained stable with the storage time. This illustrated that JLQKL_50_ could act as a dispersant to prevent cellulase from aggregating.

According to the above experimental results and analyses, a potential mechanism to enhance the enzymatic hydrolysis of corn stalk by adding JLQKL_50_ is proposed. JLQKL_50_ contains a crosslinked lignin backbone and branched cationic groups, which are hydrophobic and electropositive, respectively. It could adsorb on the hydrophobic and electronegative lignin in the substrate through hydrophobic interaction and electrostatic interaction to make the substrate hydrophilic and electropositive. Meanwhile, the cellulase also becomes electropositive because of the adsorption of JLQKL_50_ through hydrophobic interaction. Thus, the nonproductive adsorption of cellulase on lignin in the substrate is reduced by electrostatic repulsion. In addition, the dispersity and stability of cellulase are improved by JLQKL_50_. Due to the aforementioned reasons, the cellulase and cellulose in the substrate could have more opportunities to interact with each other so that the enzymatic hydrolysis efficiency can be increased.

## 4. Conclusions

In summary, new cationic kraft lignin (JLQKL_50_) with good water solubility was successfully synthesized by quaternization combined with crosslinking using KL as a raw material, CHPTMAC as a cationic modifier, and PEGDGE as a crosslinker. There was a 11.62% increase rate in the enzymatic hydrolysis efficiency of corn stalk at solid content of 10% (*w*/*v*) after 24 h when the dosage of JLQKL_50_ was fixed at a concentration of 2 g/L. With this dosage of JLQKL_50_, the enzymatic hydrolysis efficiency after 72 h reached 91.11% and 97.92% when the enzyme loading was 10 FPU/g and 15 FPU/g, respectively. The enzymatic hydrolysis system containing JLQKL_50_ could present high efficiency (higher than 70%) at a wide pH range (at least 4.5–6.0) and temperature range (at least 45–55 °C). The ranges were wider than those of the control without JLQKL_50_. The cellulase activity with the protection of JLQKL_50_ under intense agitation remained at 82.6%, which was much higher than that (70.3%) without JLQKL_50_. The promotion effect of JLQKL_50_ on enzymatic hydrolysis is likely due to the reduction in the nonproductive adsorption of cellulase on substrate lignin and the improvement in the longevity, dispersity, and stability of cellulase.

## Data Availability

The data presented in this study are available on request from the corresponding author.

## Supplementary Materials

The following supporting information can be downloaded at: https://www.mdpi.com/article/10.3390/polym15091991/s1, Figure S1: Schematic diagram of the synthesis of JLQKL_50_; Figure S2: Zeta potential of KL and JLQKL_50_ under different pH conditions; Table S1: Elemental composition of KL and JLQKL_50_.
