# Supplementary material for: Employing Cationic Kraft Lignin as Additive to Enhance Enzymatic Hydrolysis of Corn Stalk
Source: Polymers (Basel). 2023 Apr 23;15(9):1991. doi: 10.3390/polym15091991 (PMC10180774; doi:10.3390/polym15091991)
Supplement: Supplementary file 1 [file polymers-15-01991-s001.zip › polymers-2297416-supplementary.pdf]

Employing Cationic Kraft Lignin as Additive to Enhance Enzymatic Hydrolysis of Corn Stalk

Jingliang Xu [1,2], Huihua Li [1], Md. Asraful Alam [1], Gul Muhammad [1], Yongkun Lv [1], Anqi Zhao [3], Shen Zhang [1] and Wenlong Xiong [1,2,*]

[1] School of Chemical Engineering, Zhengzhou University, Zhengzhou 450001, China
[2] Henan Center for Outstanding Overseas Scientists, Zhengzhou 450001, China
[3] School of Life Sciences, Zhengzhou University, Zhengzhou 450001, China
* Correspondence: xiongwenlong@zzu.edu.cn

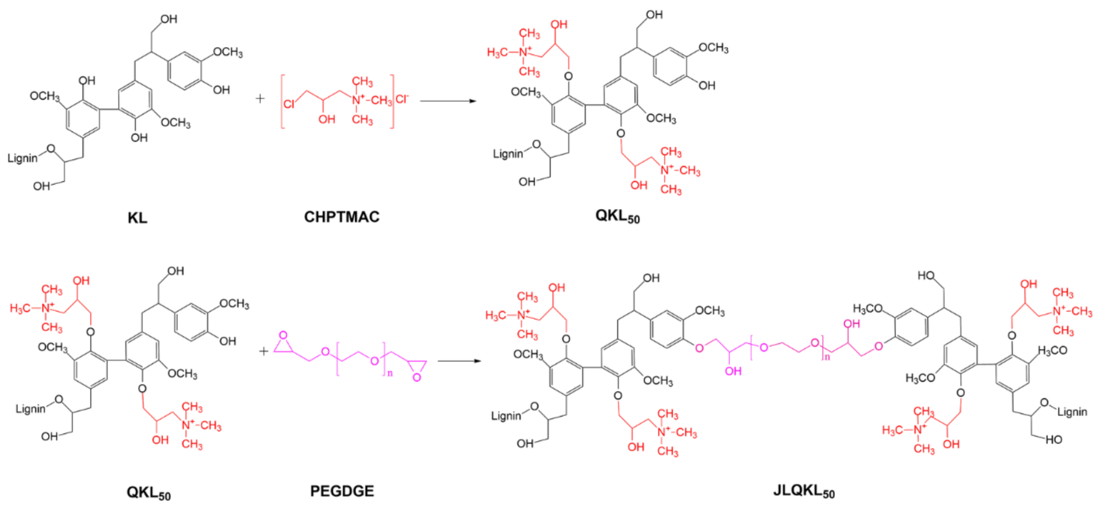

Figure S1. Schematic diagram of the synthesis of $JLQKL_{50}$

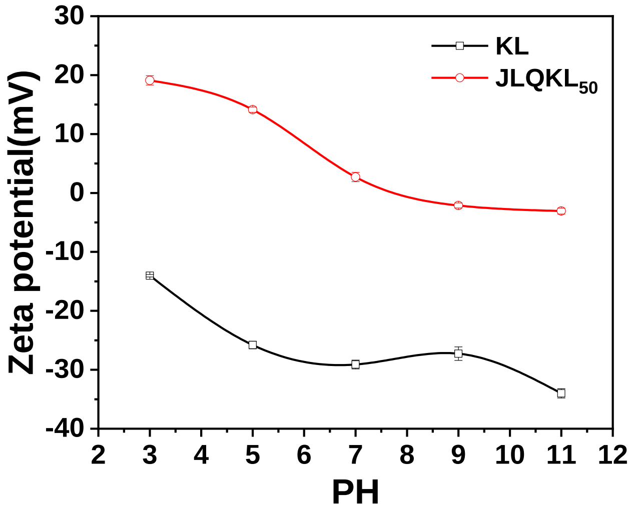

Figure S2. Zeta potential of KL and $JLQKL_{50}$ under different pH conditions

Table S1.

Elemental composition of KL and $JLQKL_{50}$

	N (%)	C (%)	H (%)	S (%)
KL	0.28	63.845	5.459	1.1565
$JLQKL_{50}$	2.165	56.06	7.1875	1.4455
